# In Vitro Reconstruction of *Xenopus* Oocyte Ovulation

**DOI:** 10.3390/ijms20194766

**Published:** 2019-09-26

**Authors:** Alexander A. Tokmakov, Yuta Matsumoto, Takumi Isobe, Ken-Ichi Sato

**Affiliations:** Faculty of Life Sciences, Kyoto Sangyo University, Kamigamo-motoyama, Kita-ku Kyoto 603-8555, Japan; hayasinosei@yahoo.co.jp (Y.M.); dortmundt-23@docomo.ne.jp (T.I.)

**Keywords:** oocytes, in vitro ovulation, maturation, MAP kinase, matrix metalloproteinases, *Xenopus laevis*

## Abstract

Progesterone is widely used to induce maturation of isolated fully grown oocytes of the African clawed frog, *Xenopus laevis*. However, the hormone fails to release oocytes from the layer of surrounding follicle cells. Here, we report that maturation and follicle rupture can be recapitulated in vitro by treating isolated follicular oocytes with progesterone and low doses of the matrix metalloproteinase (MMP), collagenase, which are ineffective in the absence of the steroid. Using this in vitro ovulation model, we demonstrate that germinal vesicle breakdown (GVBD) and oocyte liberation from ovarian follicles occur synchronously during ovulation. Inhibition of the MAPK pathway in these experimental settings suppresses both GVBD and follicular rupture, whereas inhibition of MMP activity delays follicular rupture without affecting GVBD. These results highlight importance of MAPK and MMP activities in the ovulation process and provide the first evidence for their involvement in the release of oocytes from ovarian follicles in frogs. The in vitro ovulation model developed in our study can be employed for further dissection of ovulation.

## 1. Introduction

Oocytes of most vertebrate species, including frogs, reside and grow in the ovaries while arrested at the diplotene stage in prophase of the first meiotic division. Follicular oocytes are surrounded by the follicle envelope, which consists of several layers, such as theca/epithelial layer, granulose cell layer, and vitelline envelope. The fully grown diplotene oocytes are fertilization incompetent. They display low activity of the key meiotic regulators, such as the maturation-promoting factor (MPF, a complex of cyclin B and Cdk1 kinase) and the cytostatic factor (CSF, a multicomponent system comprising the meiotic protein kinase Mos and the MAPK pathway) [1,2]. The oocytes acquire fertilization competence in the process of meiotic maturation, where they progress through the meiotic cell cycle and arrest again before fertilization. The timing of the second meiotic arrest in the meiotic cell cycle differs between species. In frogs, mature oocytes, also called eggs, arrest in metaphase II with high activities of MPF and CSF. Maturation and release of oocytes from their preovulatory follicles are initiated by a steroidogenic signal. In amphibians, it is established that the pituitary luteinizing hormone (LH) stimulates the release of steroid hormones by steroidogenic ovarian follicle cells (granulose cells) of the follicle envelope. Injection of human chorionic gonadotropin (hCG), which shares structural similarity with LH and activates the same LHCGR receptor, is ordinarily used for induction of ovulation in *Xenopus laevis* frogs. The increased production of the three main steroids, such as progesterone (PG), testosterone, and estradiol, was reported to occur after stimulation of frog follicles by gonadotropins [3,4,5]. PG is suggested to be the major physiological mediator responsible for triggering maturation of frog oocytes because its addition to the isolated defolliculated oocytes at submicromolar and low micromolar concentrations induces meiotic maturation [6,7] and because its intra-oocyte level increases abruptly to micromolar concentrations during meiosis re-entry [8]. This steroid is widely used to induce in vitro oocyte maturation in *Xenopus* models. It should be noted, however, that frog oocytes can be induced to maturation in response to multiple steroid hormones [9,10]. Evidence has been presented that androgens might be the primary steroids produced by the *Xenopus* ovary to promote oocyte maturation via classical androgen receptor [11,12]. Moreover, it is also possible that both types of steroids are involved in meiotic maturation of *Xenopus* oocytes, considering high oocyte levels of CYP17, an enzyme converting progestins to androgens [13,14].

The entire process of follicle responses to hormonal stimulation, which produces mature, follicle-free, fertilization-competent oocytes, is termed “ovulation”. Of note, in some studies the term “ovulation” is used in a narrow sense, only for oocyte liberation from the follicles and release into the oviduct. In the present article, we keep to the former, generally accepted concept of ovulation that engages, as the major events, oocyte maturation and oocyte release from ovarian follicles. Although these events appear to be highly coordinated and interrelated, they can be completely dissociated. Dissociation of the two processes was demonstrated by the in vitro exposure of *Rana pipiens* ovarian fragments to PG or corticosterone. The steroids were found to stimulate fully grown amphibian oocytes in ovarian fragments to undergo germinal vesicle breakdown (GVBD) and maturation in the complete absence of follicular rupture [15]. Defolliculated frog oocytes too can undergo meiotic maturation after PG addition [6,7]. Moreover, isolated follicular oocytes, regardless of removal of any of follicle wall layers, such as surface epithelium, theca, or follicle cells, mature but do not ovulate following exposure to PG [16]. It was found that steroid-mediated maturation is transcription-independent, as it is unaffected by actinomycin D, however, de novo transcription seems to be required for ovulation, which is greatly inhibited by the drug [17,18]. At present, the causality between the two major processes of ovulation is not understood.

To facilitate dissection of the ovulatory process, several in vitro ovulation models have been developed in mammals. Besides, an attempt to engineer the whole ovarian cell cycle using in vitro follicle culture of murine follicles was reported recently [19]. These studies helped to establish the mechanism of follicular rupture during ovulation, and they also revealed the important role of proteolytic enzymes in this process. Specifically, the members of matrix metalloproteinase (MMP) family and tissue inhibitors of MMPs were implicated in follicle rupture during ovulation in mammals [20,21,22]. It was suggested that follicle rupture occurs as a result of restricted proteolysis at the apical region of follicles. In addition, in vitro ovulation models developed for the medaka fish *Oryzias latipes* allowed identification of the hydrolytic enzymes responsible for oocyte liberation during medaka ovulation. It was demonstrated that the follicle rupture during ovulation involves cooperation of the MMP tissue inhibitor with at least three different MMPs [23,24,25]. In frogs, in vitro maturation and ovulation of *Rana pipiens* and *Rana dybowskii* oocytes was observed in the ovarian fragments and isolated follicles treated with homologous pituitary extracts [26,27,28]. It was noted that the efficiency of pituitary preparations varied considerably, exhibiting seasonal dependence [28]. In addition, various steroids were found to be effective in inducing in vitro maturation of isolated frog oocytes. However, the hormones failed to promote follicular rupture [15,16].

In the present study, to further delineate the ovulatory process with the purpose of better understanding the ovarian life cycle, we developed a new in vitro ovulation model using isolated fully grown follicular oocytes of the African clawed frog *Xenopus laevis*. Frog oocytes have been widely used in studies of maturation and meiotic progression. In fact, most of the control mechanisms that operate in meiosis, including MPF and CSF, were first established in frogs. However, the entire phenomenon of ovulation has not been studied extensively in this biological model due to, in part, difficulty of dissecting ovulation in living animals and lack of reliable in vitro reconstitution of this process. The relationship between steroid-initiated maturation and follicle rupture was not investigated in detail, and the involvement of proteolytic enzymes in follicle rupture was not demonstrated. Thus, in the present work we introduce a new in vitro ovulation model that can reproduce, simultaneously, both meiotic oocyte maturation and follicle rupture in the isolated ovarian follicles of *Xenopus laevis*. Using this model, we demonstrate for the first time that MAPK and MMP activities are involved in follicular rupture during ovulation of frog oocytes.

## 2. Results

### 2.1. PG and Gonadotropin Induce In Vitro Oocyte Maturation without Follicle Rupture

**Figure 1:**
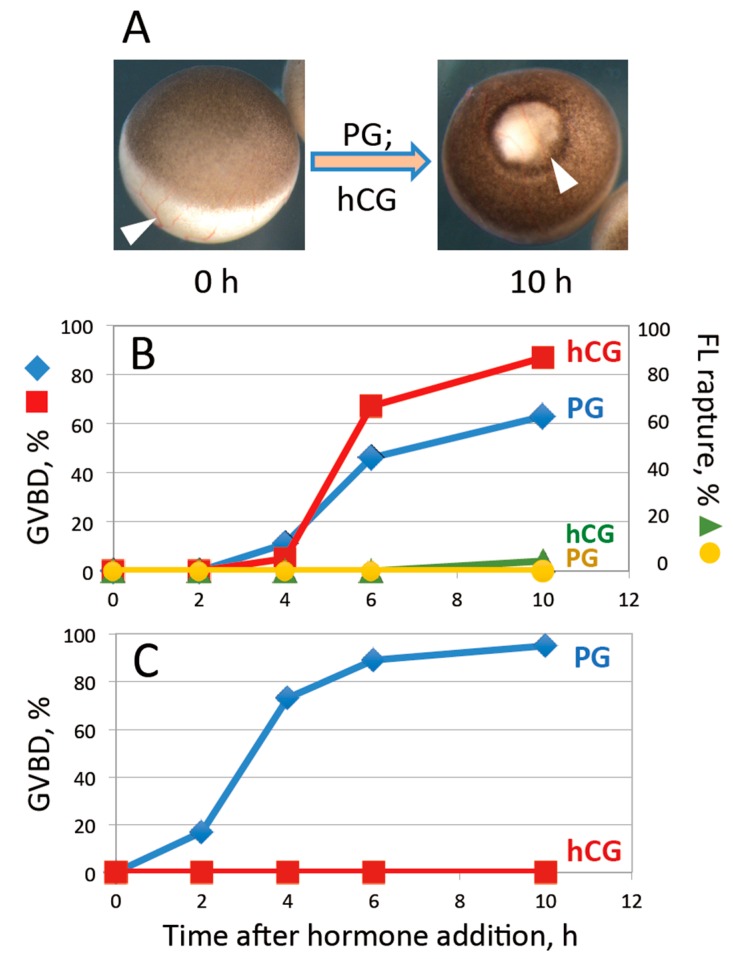
Effects of progesterone (PG) and human chorionic gonadotropin (hCG) on *Xenopus* follicular oocytes in vitro. (**A**) Morphology of isolated stage VI follicles before and after hormonal treatment. The white arrowheads in the panel point to the thin blood capillaries visible in the follicle layer. (**B**) Time course of germinal vesicle breakdown (GVBD) and follicle rupture in the hormone-treated oocytes. (**C**) Effects of the ovulation hormones on defolliculated oocytes. To remove the follicle layer, oocytes were treated with 5 mg/mL collagenase for 3 h before hormone administration. The experiment was repeated with five batches of follicular oocytes obtained from different animals. The results of a single batch experiment are presented in the figure.

Fully grown oocytes, surgically isolated from the frog ovary, are surrounded by a layer of follicle cells (Figure 1A). Both PG and hCG could induce oocyte maturation in vitro with similar efficiency and time course, as judged by occurrence of GVBD (Figure 1B). However, neither of the hormones could promote follicle rupture and oocyte release from the layer of follicle cells in the process of maturation (Figure 1A,B). In this case, when the hormones were added to defolliculated oocytes, only PG was able to induce maturation, whereas hCG was completely ineffective (Figure 1C). This result agrees well with the notion that physiological maturation of *Xenopus* oocytes in the ovaries is triggered by PG, which is released by surrounding follicle cells in response to pituitary LH. Of note, induction of maturation by PG is much faster and more efficient in defolliculated oocytes than in the follicles (Figure 1B,C), suggesting that the follicle layer may not be well permeable for the hormone (see “Discussion” for more details).

### 2.2. Low Concentration of Collagenase in the Incubation Medium Enables Hormone-dependent Follicular Rupture

**Figure 2:**
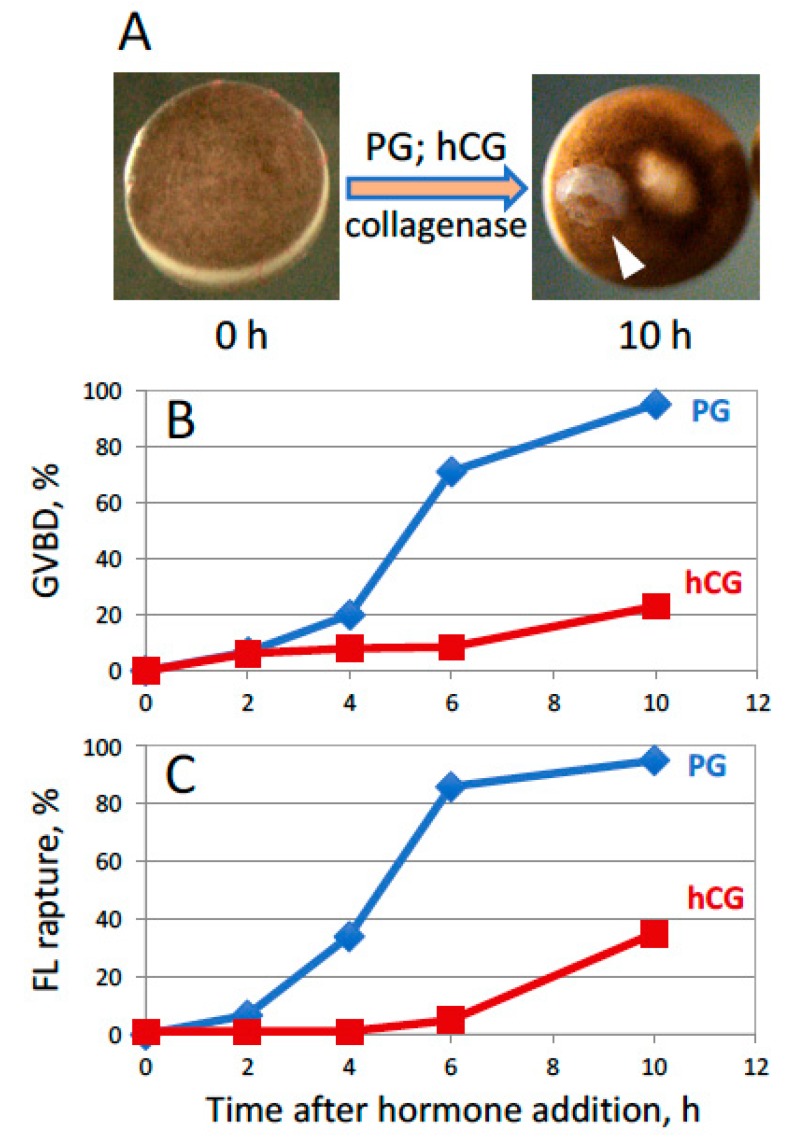
Effects of PG and hCG on the follicular oocytes in the presence of low concentrations of collagenase. (**A**) Morphology of isolated follicles before and after hormonal treatment in the presence of 50 µg/mL collagenase. A white arrowhead in the panel points to a remnant of the follicle layer that is still attached to the oocyte after follicle rupture. (**B**,**C**) Time courses of GVBD and follicle rupture, correspondingly. The experiment was repeated with five batches of follicular oocytes obtained from different animals, and the results of a single batch experiment are presented.

The fact that the ovulation hormones could not promote follicle rupture suggested that some additional factor(s) are involved in this process. We hypothesized that MMP activity might be involved in defolliculation, as it has been reported in other animal species. To reconstitute this feature of ovulation, collagenase, an enzyme of the MMP family that is commonly used to defolliculate frog oocytes, was employed. For this purpose, collagenase at a low concentration of 50 µg/mL was included continuously after hormone administration in the oocyte incubation medium. These conditions correspond to the situation when MMP enzymatic activity is mildly elevated in the ovary for a long period of time during ovulation. It was found that collagenase effectively empowered hormone-dependent follicle rupture and mildly boosted oocyte maturation in PG-treated follicles (Figure 2). Notably, the rates of hormone-dependent GVBD and follicle rupture were considerably lower in hCG- than in PG-treated follicles (Figure 2B,C). Spontaneous defolliculation, which occurred in the absence of GVBD, was rarely observed at this concentration of collagenase, however, a higher concentration of the enzyme (500 µg/mL) increased substantially the rate of spontaneous defolliculation (data not shown). Considering that spontaneous defolliculation is hormone-independent and it should be kept to a minimum, the low concentration of collagenase (~50 µg/mL) was regarded as optimal. Altogether, our findings indicate that collagenase promotes follicle rupture and facilitates maturation in PG-treated oocytes, but it exerts an adverse effect on the steroid-producing function of follicle cells treated with hCG. Therefore, in the following experiments, in vitro ovulation was triggered exclusively by PG.

### 2.3. Short-term Pretreatment of Follicles with Collagenase Enables Hormone-Dependent Follicular Rupture

**Figure 3:**
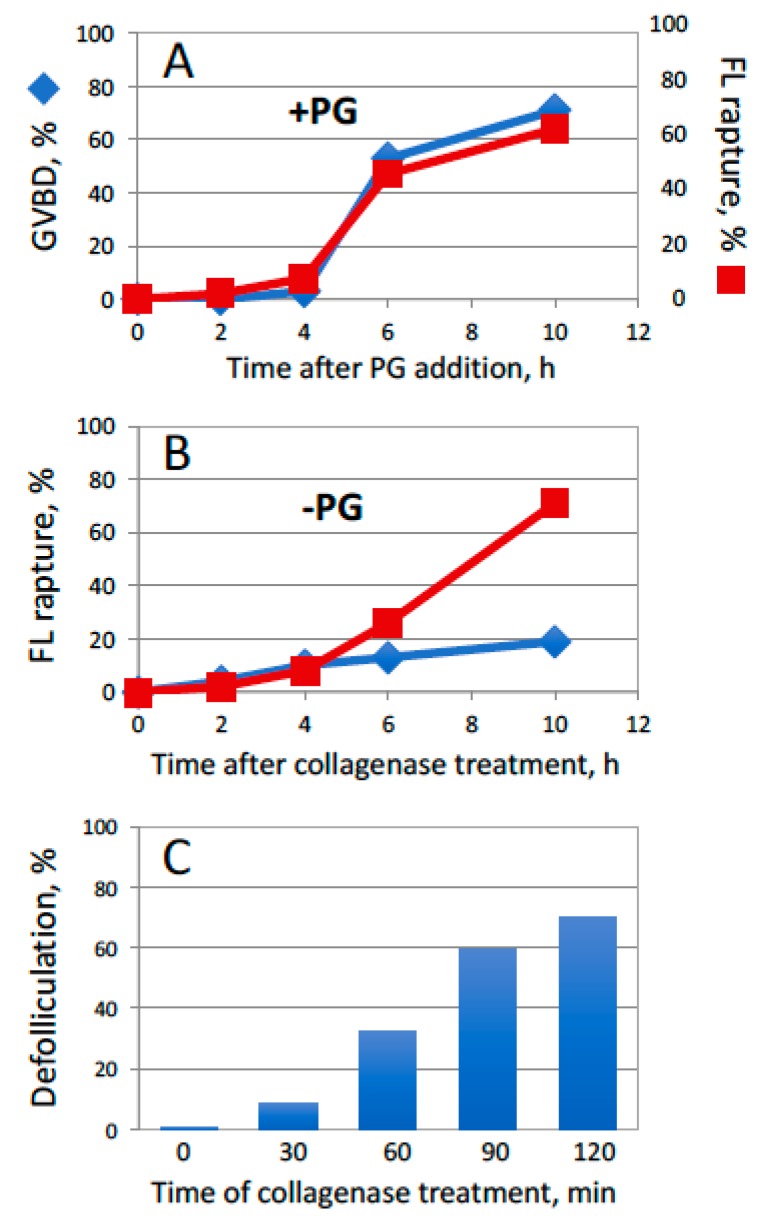
Effects of PG on the follicular oocytes pretreated with collagenase. Isolated follicles were pretreated with 5 mg/mL collagenase for 30 min before PG administration. (**A**) Time courses of GVBD and follicle rupture in the PG-treated oocytes; (**B**) time courses of spontaneous hormone-independent defolliculation in the follicles pretreated with collagenase for 30 min (blue color) or 120 min (red color); (**C**) time dependency of defolliculation in the presence of 5 mg/mL collagenase. The experiment was repeated with five batches of follicular oocytes obtained from different animals, and the results of a single batch experiment are presented.

To reinforce the suggestion that an MMP-like activity might be involved in follicle rupture during *Xenopus* oocyte ovulation, another plausible mode of enzyme’s action was investigated. It corresponds to the scenario when MMP activity is greatly elevated for a short period of time. It was found that pretreatment of isolated follicles with 5 mg/mL collagenase for 30 min, which was not sufficient to completely remove the follicle layer, could empower PG-dependent follicle rupture. This process was accompanied by oocyte maturation (Figure 3A). The rate of spontaneous hormone-independent defolliculation, which occurred in the absence of oocyte maturation, was quite low in the follicles pretreated with collagenase for 30 min (Figure 3B). However, a longer pretreatment with the enzyme produced significant numbers of defolliculated oocytes and it also increased the rates of spontaneous PG-independent defolliculation (Figure 3B,C). The collagenase treatment alone did not promote GVBD in the absence of PG (data not shown).

### 2.4. Timing of Maturation and Follicular Rupture During In Vitro Ovulation

**Figure 4:**
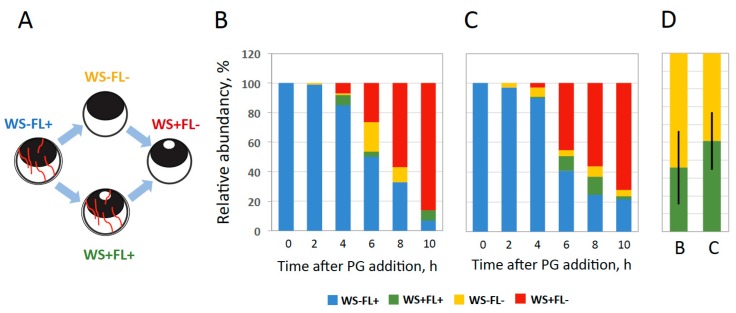
Synchronization of GVBD and follicular rupture during in vitro ovulation. (**A**) Two possible sequences of events during ovulation. The four distinctive morphological types can be observed during this process: oocytes without a white spot surrounded by follicle cells (WS−FL+), oocytes with a white spot and follicle cells (WS+FL+), oocytes without a white spot and follicle cells (WS−FL−), and oocytes with a white spot and without follicle cells (WS+FL−). (**B**) Ratios of the oocyte phenotypes in the PG-treated follicular culture preincubated for 30 min with 5 mg/mL collagenase; (**C**) The phenotype ratios in the follicle culture treated with the hormone in the presence of 50 µg/mL of collagenase. (**D**) Relative frequencies of the two intermediate phenotypes WS+FL+ and WS−FL− under the conditions described for panels (**B**,**C**). The experiment was repeated with five batches of follicular oocytes obtained from different animals, and the results of a single batch experiment are shown in panels (**B**,**C**). Bars in panel (**D**) represent SD values of the mean obtained in five experiments.

Although a mild collagenase treatment was shown to promote hormone-dependent release of oocytes from the follicle layer (Figure 2 and Figure 3), the relative timing of oocyte maturation and follicular rupture remained unclear. It is thought that the two processes occur simultaneously in vivo because mature oocytes are not retained in the frog ovary during ovulation. In our in vitro study, progressing of meiotic maturation was judged by appearance of a white spot (WS) on the animal hemisphere of oocytes, reflecting GVBD, and occurrence of follicular rupture was defined by the absence of the follicle layer (FL). Theoretically, two alternative scenarios could occur during ovulation, as indicated in Figure 4A. To distinguish between them, the four morphologically different types of cells were counted during hormone-induced ovulation in vitro. They included oocytes without a white spot surrounded by follicle cells (WS−FL+), oocytes with a white spot and follicle cells (WS+FL+), oocytes without a white spot and follicle cells (WS−FL−), and oocytes with a white spot and without follicle cells (WS+FL−). The WS−FL+ phenotype characterizes the original oocyte population, whereas the WS+FL− phenotype corresponds to ovulated mature eggs. The presence and relative frequency of the intermediate phenotypes, WS+FL+ and WS−FL−, reflects the faster occurrence of either GVBD or follicular rupture, correspondingly. Remarkably, both intermediate phenotypes could be observed in collagenase pretreated follicles (Figure 4B) and the follicles treated with PG in the presence of low concentrations of collagenase (Figure 4C). The relative frequencies of the two intermediate phenotypes observed during the entire ovulation process were close in both cases (Figure 4D), indicating that GVBD and follicular rupture occur virtually simultaneously during in vitro ovulation. Notably, the phenotype frequencies varied greatly from experiment to experiment (Figure 4D), reflecting differences in individual oocyte batches.

### 2.5. Follicular Rupture Is Delayed in the Presence of an MMP Inhibitor

**Figure 5:**
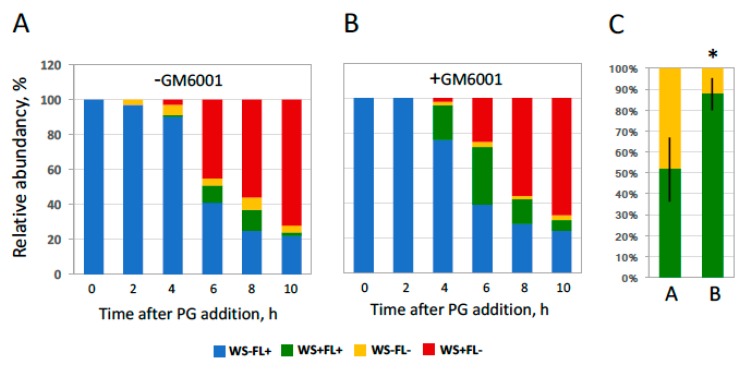
Effect of matrix metalloproteinase (MMP) inhibition on oocyte maturation and defolliculation. (**A**,**B**) Ratios of the specific oocyte phenotypes, as designated in the legend to Figure 4, in the follicular culture preincubated for 30 min with 5 mg/mL collagenase and treated with PG in the absence or presence of an MMP inhibitor, respectively. The inhibitor was added to the follicular oocytes at a final concentration of 50 µM at the time of PG administration. (**C**) Relative frequencies of the two intermediate phenotypes, WS+FL+ and WS−FL−, under the conditions described for panels (**A**,**B**). Bars in panel (**C**) represent SD values of the mean obtained in four experiments. An asterisk in panel C indicates statistical significance in the phenotype ratio between A and B (*p* < 0.05).

The data presented in Figure 2 and Figure 3 suggest that MMP activity might be involved in *Xenopus* oocyte ovulation. To verify this suggestion, next we investigated the effect of a wide-specificity MMP inhibitor GM6001 in the developed model of in vitro ovulation. It was found that the inhibitor delayed follicle rupture, as it could be concluded from the significantly increased proportion of the WS+FL+ phenotype observed during the entire in vitro ovulation process (Figure 5). However, the overall outcome of ovulation was not affected by MMP inhibition; the resulting proportion of ovulated matured oocytes (WS+FL−) was the same (~70%) in the presence and absence of the inhibitor after 10 h of the treatment. In addition, MMP inhibition had no effect on oocyte maturation; both the time course and the resulting rate of GVBD were almost identical in the presence or absence of the inhibitor (Figure 5).

### 2.6. Inhibition of the MAPK Pathway Suppresses Both Steroid-Induced Maturation and Follicular Rupture

**Figure 6:**
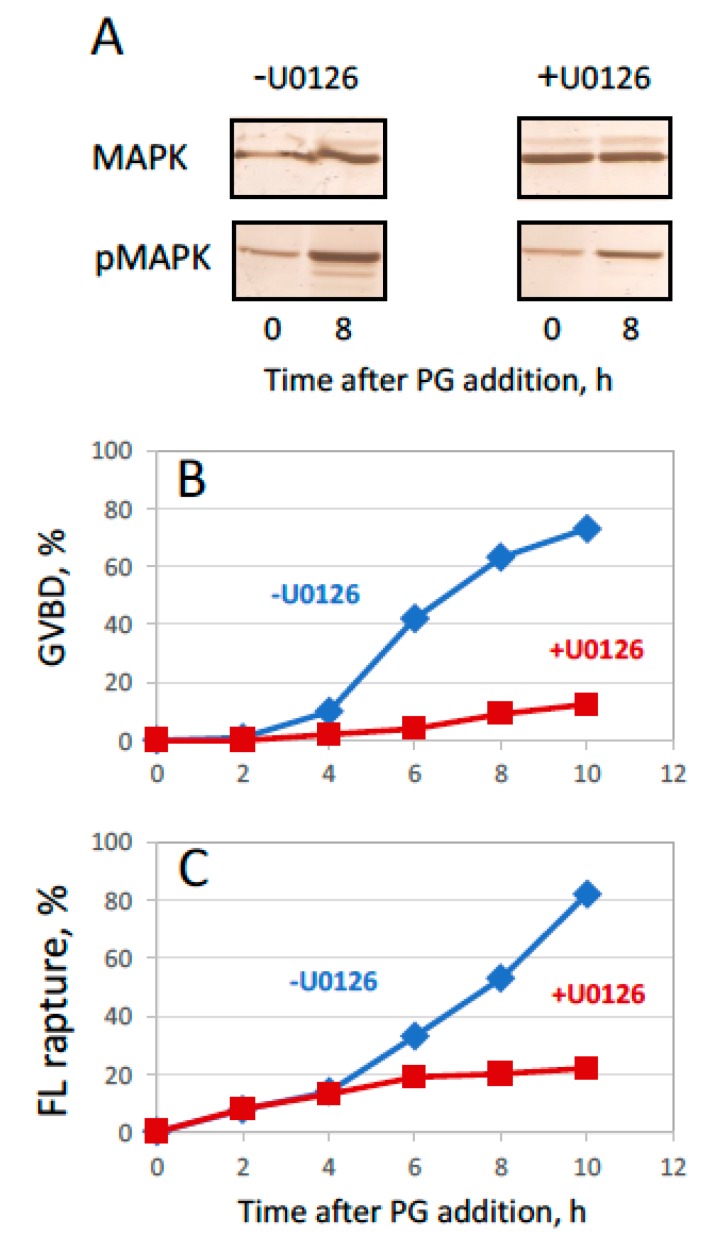
Effect of MAPK pathway inhibition on maturation and defolliculation. (**A**) Phosphorylation status of MAPK in the absence or presence of an MAPKK inhibitor during in vitro ovulation of *Xenopus* oocytes. The MAPKK inhibitor U0126 was added to the follicular oocytes at a final concentration of 50 µM at the time of PG administration. (**B**,**C**) Time courses of GVBD and follicular rupture, respectively. The experiment was repeated with four batches of follicular oocytes obtained from different animals and the results of a single batch experiment are presented.

Temporal correlation of GVBD and follicular release (Figure 2, Figure 3 and Figure 4), and the fact that follicle rupture in collagenase-treated oocytes does not occur in the absence of hormonal stimulation (Figure 2 and Figure 3), indicate that initiation of the meiotic maturation is necessary (but not sufficient) for follicle rupture. To confirm interconnection between the two main processes of ovulation, the effect of MAPK pathway inhibition was investigated in the developed model of in vitro ovulation. It was demonstrated previously that inhibition of the MAPK pathway hinders meiotic transition in *Xenopus* oocytes. We found that the specific inhibitor of MEK, U0126, greatly attenuated PG-induced MAPK activation when added to isolated follicles at 50 µM concentration (Figure 6A). Furthermore, the inhibitor effectively suppressed both oocyte maturation and follicular rupture (Figure 6B,C). However, inhibition of the MAPK pathway had no effect on spontaneous defolliculation of oocytes that occurred in the absence of PG treatment (data not shown). These results strongly suggest the existence of causal relationships between meiotic maturation and follicular release (see “Discussion” for more details).

## 3. Discussion

In the present work, we introduce a novel original model for in vitro ovulation of isolated follicular oocytes of the African clawed frog *Xenopus laevis*. This model recapitulates the two most important processes of ovulation, such as meiotic maturation and oocyte release from ovarian follicles. Thereafter, the developed ovulation model was used to delineate the occurrence of GVBD and follicular rupture during ovulation and to demonstrate involvement of the MAPK pathway and MMP activity in the ovulation process. 

The steroid hormone PG is commonly used to initiate maturation of isolated *Xenopus* oocytes. The steroid acts directly on the oocytes to induce maturation. It is established that in the frog’s body, PG is secreted in response to LH, which is structurally and functionally similar to gonadotropins, by the follicle cells surrounding the oocyte. Indeed, our study confirms that hCG can promote oocyte maturation with nearly the same efficiency as PG in the isolated oocytes surrounded by follicle layer, however it is completely ineffective when added to defolliculated oocytes (Figure 1). In contrast, PG induces maturation both in the presence and absence of follicle cells. However, considering maturation kinetics, it can be concluded that the follicle layer is not well permeable for the hormone (Figure 1B). Of note, it was reported previously that steroid efficiency to promote *Xenopus* oocyte maturation is reduced by follicle cells [8]. 

Importantly, neither PG nor hCG alone can promote follicle rupture in isolated follicular oocytes (Figure 1). On the one hand, this finding is consistent with previous studies reporting that follicular oocytes of *Rana pipiens* mature but do not ovulate following exposure to PG [15,16]. On the other hand, it could be expected that hCG would be able to initiate both maturation and follicle rupture, considering that pituitary extracts were shown to be effective in promoting these processes in vitro [26,27,28]. Moreover, in vivo ovulation of frog oocytes can be induced by hCG injection into the dorsal lymph sac. It was suggested that ovulation in vitro requires both gonadotropin priming and PG for successful follicle rupture in primates [29]. However, in our experiments simultaneous addition of the two hormones to isolated follicular oocytes of *Xenopus laevis* failed to initiate follicle rupture (data not shown), suggesting that some additional factor(s) are involved in this process. 

In the present study, we hypothesized that MMP activity might contribute to follicular rupture during frog oocyte ovulation. Previously, this activity was found to be involved in ovulation in mammals [20,21,22] and in the medaka fish [23,24,25], however, no evidence has been presented so far for its involvement in frogs. We have found that both maturation and follicle rupture can be recapitulated in vitro in the follicular *Xenopus* oocytes treated with PG and low concentrations of collagenase, an enzyme of MMP family, (Figure 2), as well as in the oocytes pretreated with collagenase shortly before PG administration (Figure 3). Importantly, either collagenase treatment was ineffective in the absence of the steroid. Using this approach, the relative timing of the two major ovulation events, such as oocyte maturation and release from the follicular layer, was delineated (Figure 4). The facts that follicular rupture occurs at about the same time as GVBD and it doesn’t occur at all in the absence of PG (Figure 3 and Figure 4), indicate that follicular rupture is coordinated with initiation of meiotic maturation. Indeed, PG was shown to be required for follicular rupture in many species. An absolute requirement for PG in follicle rupture has been documented in rodents and primates, wherein inhibition of PG synthesis or its activity prevented ovulation [30,31,32,33]. It was also found that mice lacking nuclear PG receptor do not ovulate [34]. The precise mechanism by which PG promotes follicle rupture remains unknown. There is mounting evidence for a link between PG and its regulation of MMPs and other proteases [35,36]. 

In the light of previous findings, it was natural to surmise that inhibition of PG-induced oocyte maturation might also suppress follicular rupture in our experimental settings. Therefore, we investigated the effect of MAPK pathway inhibition on oocyte ovulation in the developed in vitro model. It is well established that activity of MPF and CSF increase dramatically during steroid-induced meiotic maturation of frog oocytes. The two major meiotic regulators are embedded in a loop of positive feedback that helps to promote their mutual activation during oocyte maturation and maintain high activity during metaphase II arrest [37,38,39]. Inhibition of the MAPK pathway, which represents one of the major CSF components, was reported to suppress, or delay significantly, *Xenopus* oocyte maturation [40,41,42]. Accordingly, in our experiments, the specific inhibitor of MEK, U0126, restrained PG-induced oocyte maturation (Figure 6B). Remarkably, the inhibitor also attenuated steroid-induced follicle rupture to the same extent (Figure 6C), strongly suggesting that initiation of meiotic maturation is mandatory for follicular release. To our knowledge, this is the first evidence for involvement of MAPK activity in follicle rupture during ovulation of frog oocytes. 

Of special interest is participation of follicular MAPK in the regulation of ovulation. Phosphorylation and activation of ERK1/2 was observed in granulosa cells of different species in response to luteinizing hormone [43,44,45]. In mice, the hormone induces ERK1/2 phosphorylation in preovulatory follicles within 30 min, progressing first in mural granulosa cells, and then in cumulus cells [46]. Evidence suggests that transactivation of EGFR is important for regulating hormone-initiated activation of ERK1/2 in the follicles [47]. It was reported that in cultured mouse follicles, the MEK inhibitor U0126 blocked both hormone-induced GVBD and cumulus expansion, albeit when used at a high concentration that could possibly produce nonspecific effects [44]. Moreover, when ERK1/2 was disrupted specifically in mouse granulosa cells, oocyte maturation, cumulus expansion, and ovulation failed to occur in response to gonadotropin [48]. At present, it is not known whether MAPK becomes activated in follicle cells during ovulation in frogs. Also, the effect of targeted inhibition of the MAPK pathway in these cells was not investigated. In our experiments, the MEK inhibitor, when added to isolated follicular oocytes, should have affected both oocytic and follicular MAPK. It will be necessary to discriminate between the two pools of MAPK in the future experiments.

In addition, the results obtained in this study with the use of the new in vitro ovulation model present strong evidence the for involvement of MMP-like activity in *Xenopus* oocyte ovulation. Indeed, PG-dependent follicle rupture during in vitro ovulation only becomes possible in the presence of low concentrations of collagenase or in the follicles pretreated with collagenase for a short time (Figure 2 and Figure 3). Furthermore, oocyte release from ovarian follicles is inhibited in the presence of a wide-specificity MMP inhibitor GM6001 (Figure 5). Although, it was demonstrated previously that enzymes of the MMP family participate in the egg ovulation process in several species [20,21,22,23,24,25], no reports have been presented so far concerning the involvement of this enzymatic activity in ovulation of frog oocytes. Our study reveals for the first time that MMP activity is involved in oocyte liberation from ovarian follicles in frogs. 

At present, causality between the two major ovulation processes, meiotic maturation and oocyte release from ovarian follicles, is not understood. It is not known how the two processes are synchronized. Strong control mechanisms should be employed in animals’ bodies to coordinate meiotic maturation and follicular release of oocytes during ovulation. The facts, that all naturally ovulated eggs are matured and arrested in metaphase II with high activity of MPF and CSF, and all nonovulated oocytes that are retained in the ovary after the hormonal stimulation remain immature and arrested in prophase I with low activity of MPF and CSF, demonstrate that these control mechanisms ensure that only the follicles with maturation-competent oocytes undergo follicle rupture. Further studies are necessary to clarify how meiotic maturation and follicle rupture are coordinated during ovulation.

## 4. Materials and Methods

### 4.1. Materials

Anesthetic MS-222 and water-soluble PG were purchased from Sigma (St. Louis, MO, USA). Collagenase (280 U/mg) was obtained from Wako (Osaka, Japan) and hCG was from Teikoku Zoki (Tokyo Japan). Polyclonal anti-MAPK and anti-pMAPK antibodies were from Cell Signaling (Beverly, MA), biotinylated anti-rabbit IgG was from Vector Laboratories (Burlingame, CA, USA), and the Streptavidin Biotin Complex Peroxidase Kit was from Nacalai Tesque (Kyoto, Japan). Polyvinulidene difluoride (PVDF) membranes Immobilon were purchased from Merck Millipore (Carrigtwohill, Ireland). Protein assay CBB solution was from Nacalai Tesque (Kyoto, Japan). Specific MEK inhibitor U0126 was from Promega (Madison, WI) and MMP inhibitor GM6001 was from Calbiochem (Darmstadt, Germany). Other chemicals were obtained from Wako and Nacalai Tesque.

### 4.2. Animals and Cells

Adult female frogs of wild-type of *Xenopus laevis* were obtained from Shimidzu (Kyoto, Japan) and maintained in dechlorinated water at the ambient temperature of 21–23 °C. The experiments with the animals were conducted according to the Kyoto Sangyo University Animal Experimentation Regulations under the permission N 2018-20. Oocytes were isolated as described previously [49]. In brief, frogs were anesthetized in 2 mg/mL solution of MS-222, then ovaries were surgically removed and placed into OR-2 buffer containing 82.5 mM NaCl, 2.5 mM KCl, 1 mM CaCl_2_, 1 mM MgCl_2_, 1 mM Na_2_HPO_4_, 5 mM HEPES, pH 7.6. Ovaries were manually dissected into small clumps, extensively washed with the buffer and left for stabilization over 4 h. Undamaged fully grown follicular oocytes of stage VI were manually selected and used in the experiments. The selected cells ranged in size from 1.2 to 1.3 mm and were characterized by an unpigmented equatorial band, as described previously [50].

In vitro oocyte maturation was induced by addition of 5 µM PG or by addition of 200 U hCG per 100 mm Petri dish containing isolated oocytes of stage VI. During maturation, the oocytes were kept at the ambient temperature of 21–23 °C. Progression of maturation was monitored over 10–12 h by appearance of a white spot on the animal hemisphere of oocytes. In addition, follicle rupture was monitored under a dissecting microscope. The rupture was considered complete when most of the oocyte body protruded from the follicle layer. 

In some experiments, the abovementioned manipulations with oocytes were carried out in MMR buffer (100 mM NaCl, 2 mM KCl, 1 mM MgCl_2_, 2 mM CaCl_2_, 0.1 mM EGTA, 5 mM HEPES, pH 7.7), which was used instead of OR-2.

### 4.3. Treatment of Oocytes with Collagenase, MMP, and MEK Inhibitors

To obtain defolliculated oocytes, clumps of 50–100 oocytes were treated with 5 mg/mL collagenase (280 U/mg) in OR-2 at 23 °C for 3 h by shaking at 60 rpm. To prime follicle-enclosed oocytes for follicular rupture, the isolated follicles were preincubated with 5 mg/mL collagenase at 23 °C for 30 min. This treatment could not liberate oocytes from the follicle layer. After the treatment, undamaged follicular oocytes were selected and used in further experiments. The oocytes were extensively washed with OR-2 buffer before PG administration to completely remove the enzyme. Alternatively, collagenase at a low concentration of 50 µg/mL was included in the incubation media during PG-induced ovulation to facilitate follicular rupture. The MMP and MEK inhibitors, GM6001 and U0126, were added to isolated follicular oocytes at a final concentration of 50 µM together with PG.

### 4.4. Microscopic Observations and Image Processing

SZX16 stereo zoom microscope (Olympus, Japan) equipped with a high-frame digital microscope CCD camera DP73, CCD interface *U*-TV0.5XC-*3*, wide-angle objective SDF PLAPO 1xPF, and CellSens Standard software was used for image acquisition. The acquired images were further processed with the ImageJ software of the National Institute of Health [51], freely downloadable from https://imagej.nih.gov/ij/.

### 4.5. Immunoblotting

To obtain cytosolic cell fractions, oocytes and eggs were homogenized by pipetting in 5x fold volume of OR-2 buffer containing protease inhibitors APMSF and leupeptin, and then centrifuged at 10,000 rpm, 4 °C, for 10 min. The supernatant fractions were collected after centrifugation and heated at 95 °C for 5 min in the presence of SDS-sample buffer. Protein samples were separated by SDS PAGE using 10% polyacrylamide gels and transferred to PVDF membranes using a semidry blotting device (BioRad). The membranes were blocked with T–TBS buffer (20 mM Tris–HCl, pH 7.5, 150 mM NaCl, 0.05% Tween 20) containing 3 mg/mL bovine serum albumin. To monitor MAPK activation status, the membranes were incubated at RT for 2 h with 100-fold diluted anti-MAPK or with 200-fold diluted anti-phospho MAPK antibodies. After washing, the membranes were treated with 1000-fold diluted biotinylated anti-rabbit IgG, then with the peroxidase-conjugated Streptavidin Biotin Complex Peroxidase Kit reagent, according to the manufacturer’s manual. The immune complexes were detected by color development catalyzed by peroxidase.

### 4.6. Other Methods 

Protein content in egg cytosolic fractions was determined with the CBB protein assay. Sample absorbance was measured using a NanoDrop 1000 Spectrophotometer (Thermo Fisher, Waltham, MA). Bovine serum albumin was applied as a calibration standard. Quantified data in figures are presented as means ± SD values of four to six measurements taken in single batch experiments. All experiments were repeated with the separate batches of oocytes and eggs obtained from at least four different animals. From 50 to 100 oocytes were observed in the experiments that concerned counting specific oocyte phenotypes. The phenotype frequencies varied greatly from experiment to experiment, reflecting differences in individual oocyte batches. 

## 5. Conclusions

In sum, we developed a new in vitro ovulation model using isolated ovarian follicles of *Xenopus laevis.* The model reproduces reliably both meiotic maturation and oocyte liberation from follicles, providing an innovative experimental approach for studying ovulation in frogs. It can be especially useful for investigating mechanisms of follicular release and coordination of meiotic maturation and follicle rupture in the ovulation process. Using this model, we demonstrate for the first time that MAPK and MMP activities are involved in oocyte release from ovarian follicles during ovulation in frogs.

## Abbreviations

CBB	Coomassie Brilliant Blue
CCD	Charge-coupled device
CSF	Cytostatic factor
ERK1/2	Extracellular signal-regulated kinases ½
FL	Follicle layer
GVBD	Germinal vesicle breakdown
hCG	Human chorionic gonadotropin
LH	Luteinizing hormone
MII	Metaphase II
MAPK	Mitogen-activated protein kinase
MEK	MAPK/ERK kinase
MMP	Matrix metalloproteinase
MPF	Maturation-promoting factor
PG	Progesterone
PAGE	Polyacrylamide gel electrophoresis
SDS	Sodium dodecyl sulfate
WS	White spot
